# Usability evaluation of a clinical decision support tool for osteoporosis disease management

**DOI:** 10.1186/1748-5908-5-96

**Published:** 2010-12-10

**Authors:** Monika Kastner, Danielle Lottridge, Christine Marquez, David Newton, Sharon E Straus

**Affiliations:** 1 Department of Health Policy, Management and Evaluation, Faculty of Medicine, University of Toronto, Toronto, Ontario, Canada; 2 Department of Mechanical and Industrial Engineering, University of Toronto, Toronto, Ontario, Canada; 3 Li Ka Shing Knowledge Institute of St. Michael's Hospital, Toronto, Ontario, Canada

## Abstract

**Background:**

Osteoporosis affects over 200 million people worldwide at a high cost to healthcare systems. Although guidelines are available, patients are not receiving appropriate diagnostic testing or treatment. Findings from a systematic review of osteoporosis interventions and a series of focus groups were used to develop a functional multifaceted tool that can support clinical decision-making in osteoporosis disease management at the point of care. The objective of our study was to assess how well the prototype met functional goals and usability needs.

**Methods:**

We conducted a usability study for each component of the tool--the Best Practice Recommendation Prompt (BestPROMPT), the Risk Assessment Questionnaire (RAQ), and the Customised Osteoporosis Education (COPE) sheet--using the framework described by Kushniruk and Patel. All studies consisted of one-on-one sessions with a moderator using a standardised worksheet. Sessions were audio- and video-taped and transcribed verbatim. Data analysis consisted of a combination of qualitative and quantitative analyses.

**Results:**

In study 1, physicians liked that the BestPROMPT can provide customised recommendations based on risk factors identified from the RAQ. Barriers included lack of time to use the tool, the need to alter clinic workflow to enable point-of-care use, and that the tool may disrupt the real reason for the visit. In study 2, patients completed the RAQ in a mean of 6 minutes, 35 seconds. Of the 42 critical incidents, 60% were navigational and most occurred when the first nine participants were using the stylus pen; no critical incidents were observed with the last six participants that used the touch screen. Patients thought that the RAQ questions were easy to read and understand, but they found it difficult to initiate the questionnaire. Suggestions for improvement included improving aspects of the interface and navigation. The results of study 3 showed that most patients were able to understand and describe sections of the COPE sheet, and all considered discussing the information with their physicians. Suggestions for improvement included simplifying the language and improving the layout.

**Conclusions:**

Findings from the three studies informed changes to the tool and confirmed the importance of usability testing on all end users to reduce errors, and as an important step in the development process of knowledge translation interventions.

## Background

Osteoporosis affects over 200 million people worldwide [1], and the fractures it can cause represent a considerable financial burden to healthcare systems [2-6]. This challenge is compounded by an increasingly aging population [2,6,7], particularly since the clinical consequences of osteoporosis can significantly impair quality of life, physical function, and social interaction and can lead to admission to long-term care [4,8]. Although guidelines are available for osteoporosis disease management [9-14], patients are not receiving appropriate diagnostic testing or treatment [15-17]. One potential solution to closing these practice gaps is to use clinical decision support systems (CDSSs), which can facilitate disease management by translating high-quality evidence at the point of care. We conducted a systematic review of randomised controlled trials to determine what features of current tools may support clinical decision-making in osteoporosis disease management [18]. Findings indicated that few osteoporosis CDSSs exist and that the disease-management components of interventions were lacking in most studies [18]. Interventions consisting of reminders and education targeted to physicians and patients appeared more promising for increasing osteoporosis investigations and treatment than did single-component or single-target interventions. Findings from the systematic review and input from clinicians and experts in information technology and human-factors engineering were used to develop a conceptual model of an osteoporosis tool. This model was qualitatively explored in a series of focus groups to determine how physicians perceived this conceptual model and which key features, functions, and evidence were needed to transform it into a functional prototype [19].

The resulting prototype tool is targeted to both physicians and patients and consists of three components: (1) an electronic osteoporosis Risk Assessment Questionnaire (RAQ) completed by eligible patients on a tablet PC in the clinic examination room; (2) a paper-based, Best Practice Recommendation Prompt (BestPROMPT) outlining appropriate osteoporosis disease-management recommendations for use by physicians at the point of care; and (3) a paper-based, Customised Osteoporosis Educational (COPE) sheet given to patients at the end of their physician visit. The first component of the tool (*i.e*., the RAQ) is designed so it can be completed on a tablet PC by eligible patients (men ≥65 years and women ≥50 years of age) in a clinic examination room during the 5- to 15-minute waiting period prior to the actual physician visit. Completion of the RAQ in the examination room provides privacy for patients and the ability to use the audio support feature of the tool. Patients can listen to the questions out loud (default) or turn off the sound at any time during the questionnaire. Once the questionnaire is completed, RAQ responses are processed using a decision algorithm programmed into the tablet PC, which automatically generates two paper-based outputs using a wireless printer: one for the physician (*i.e*., the BestPROMPT sheet) and one for the patient (*i.e*., the COPE sheet). The BestPROMPT provides a summary of the patient's RAQ responses, a section outlining appropriate osteoporosis disease-management recommendations (*e.g*., to initiate bone mineral density testing or osteoporosis medications such as bisphosphonates), and a graph to plot the patient's 10-year absolute fracture risk. These features were designed so that physicians would be able to use this information with their patients at the point of care. The COPE sheet summarizes patients' osteoporosis risks according to their RAQ responses and provides a section outlining osteoporosis information customised to their identified risks (*i.e*., an explanation of what each risk factors means, and what they can do about them).

Although information technology or CDSSs, such as the osteoporosis tool, can address important barriers to clinical practice and may enhance the safety, quality, and patient-centeredness of care while increasing efficiency [20,21], there is an increasing body of evidence showing unanticipated and undesired consequences to implementation of these systems [22-26]. Without careful consideration of system design, function, and end-user perspectives, these systems can fail if rushed to become an integral part of healthcare systems and practices either during rigorous evaluation or after implementation of such interventions [27]. If information technology systems are integrated without evaluating how they might impact end users or their existing workflow, they have the potential to be ineffective, function poorly, and result in medical or technology-induced errors [22,23]. Usability testing is an investigation of the human-computer interaction--to provide practical feedback on the design of computer systems and user interfaces and provide information about the process of using a system to characterize decision-making, reasoning skills, and the information-processing needs of participants as they perform representative tasks that require complex information processing [28-30]. Another important consideration in the prototype development process is iterative systems analysis, which involves the evaluation of the system during the design phase, followed by further cycles of redesign and testing. These evaluations are needed to ensure that the needs of end users are considered over what researchers and designers might perceive as important user requirements. Importantly, iterative analysis is needed before a system is ever considered for implementation in clinical practice [31].

The objectives of the current study were to conduct a usability evaluation of the three components of the osteoporosis tool to assess how well the prototype meets functional goals (features, format, and interface) and usability needs (outcome impact goals and end users' requirements and information needs) and to determine end users' perceptions of the facilitators and barriers to using the prototype at the point of care.

## Methods

To determine if the osteoporosis prototype meets the usability needs of all end users, a usability study was planned for each component of the tool: usability study 1 (the BestPROMPT); usability study 2 (the RAQ), and usability study 3 (the COPE sheet). All three studies were designed according to the usability framework described by Kushniruk and Patel [28] because it promotes an evidence-based approach to deriving knowledge and is regarded as the most useful method for testing usability in the medical context [32,33]. It was anticipated that the osteoporosis tool would be changed iteratively throughout the usability evaluation studies, retested and evaluated, and a final modification made once the desired functionality and usability were achieved.

All usability studies were approved by the University of Toronto and St. Michael's Hospital research and ethics boards, and a written informed consent was obtained from all participants. All studies consisted of 30- to 60-minute, one-on-one sessions with an experienced moderator using a standardised, structured worksheet combined with a semistructured discussion using open-ended questions to evaluate each tool component. Participants were encouraged to 'think aloud' and verbalise their thoughts about the component being tested. The target sample size for each study was five to eight participants because evidence indicates that 70% of severe usability problems can be uncovered within the first five users and up to 85% by the eighth user, after which the yield of identified problems tends to drop and is also less significant [28,34].

### Usability study 1: evaluation of the BestPROMPT sheet

The first study was conducted with full-time family physicians and general internal medicine specialists in the greater Toronto area between May and September 2008. Physicians were randomly selected from the College of Physicians and Surgeons of Ontario database using a standardised faxed recruitment letter. To reach the target sample size of eight participants, purposive sampling from the St. Michael's Family Practice Unit in Toronto was required. Population exclusion criteria were general internists who saw patients in a subspecialty practice that excluded the possibility of seeing patients with osteoporosis.

Usability sessions were designed to evaluate the BestPROMPT sheet with relevant end users for appropriate content and format and to include tasks that would be representative of the real uses of the sheet. This involved showing physicians how the BestPROMPT sheet is generated so that potential barriers to using it at the point of care can be addressed in the context of participants' own workflow. To achieve this, the moderator simulated a patient at risk for osteoporosis and completed the RAQ on a tablet PC, which the physician participant observed. Using a structured worksheet, the BestPROMPT copy that was generated during this exercise was used in the second part of the usability session to elicit feedback on format (*e.g*., font, spacing), readability, and understandability using a five-point Likert scale. Open-ended questions were used to probe what participants found the most/least useful about the BestPROMPT and the barriers to using the sheet at the point of care; we also included a validated, 10-item System Usability Scale [35] to assess the subjective usability of the tool.

### Usability study 2: evaluation of the RAQ

The second study was conducted with patients at risk for osteoporosis (men ≥65 years of age and postmenopausal women) between October and December 2008. Patients were selected purposively from the patient population of one family physician at the St. Michael's Family Practice Unit until at least five to eight patients (per input device) were tested or usability problems were eliminated. To maximize the number of eligible patients to be recruited, sessions were planned with patients immediately following their family physician visit.

Usability sessions were designed to evaluate the RAQ with its relevant end users (*i.e*., patients at risk for osteoporosis) for appropriate content, format, navigation, and input device (stylus, mouse and keyboard, or touch screen). The moderator used a standardised, pilot-tested script and worksheet for the sessions, which included tasks that would be the most representative of the real uses of the RAQ. A goal for these sessions was to ensure that the RAQ could be completed by participants with little to no assistance from the moderator (*i.e*., to simulate what might be expected in real practice). The usability sessions consisted of three parts: In part 1, the moderator documented observed events as participants completed each RAQ question. This was supplemented by an embedded program, which generated a timed log of each tap/click/touch to enable the calculation of the time it took to complete the RAQ and frequency of incidents and data entry errors. The incident log was developed based on the critical incidence technique pioneered by Flanagan *et al*. [36], which can provide an objective assessment of events that make the difference between success and failure (*i.e*., the critical incident) [36]. We defined an incident in terms of its negative impact: a problem or error according to two levels of severity (critical or general). A critical incident was defined as a problem that completely halted the normal functioning of the RAQ (*e.g*., unable to initiate the questionnaire), whereas a general incident could occur within one session or across sessions but did not inhibit the completion of the RAQ (*e.g*., mis-tapping of a button, activating the 'Warning' window). Incident types were classified as navigational, interface, technical, input-device related, question to moderator, or other. General incidents occurring at least two times within one or across sessions were elevated to critical status. Immediate changes were made only for critical incidents. In the second part of the usability session, observed critical incidents were used as memory probes to clarify the problem and to identify what influence the incident had on the interaction with the system. The last part of the session consisted of a series of semistructured, open-ended questions about the format, interface, features, and content of the RAQ and what participants liked/disliked about the questionnaire.

### Usability study 3: evaluation of the COPE sheet

The third study was conducted with patients at risk for osteoporosis in December 2008. Participants were selected purposively from the same family physician's patient population as used in usability study 2 until at least five to eight patients were recruited or usability problems were eliminated. Usability sessions were designed to evaluate the COPE sheet with its relevant end users (*i.e*., patients at risk for osteoporosis) for appropriate content and format. The sessions consisted of two parts: In part 1, participants were asked to complete the RAQ so they could observe how the COPE sheet is generated. This process enabled testing whether the decision algorithm accurately translated the response inputs from the RAQ into the educational content of the COPE sheet. In part 2, the moderator conducted a semistructured interview with participants to explore their understanding of the COPE sheet, what they might do if they had any unanswered questions about their osteoporosis risks, and if they might consider discussing the sheet with their physician. The moderator also asked participants to rate the readability, understandability, and format of the COPE sheet using a verbal five-point Likert scale.

### Data collection and analysis

All usability sessions were audiotaped and transcribed verbatim. Usability study 2 was also videotaped to observe users' physical behaviour as they interacted with the RAQ. Data collection and analysis consisted of a combination of qualitative analysis to assess the effect of technology on participant reasoning and decision-making, and quantitative analysis to assess data from the demographic questionnaire, System Usability Scale, critical incident log sheet, and Likert-type questions.

### Qualitative data

Qualitative content analyses were guided by the constant comparative method of grounded theory methodology [31] and verbal protocol-analysis techniques [28,29]. Audio and video data were coded from transcripts using a process of open, axial, and selective coding [37,38] using NVivo 8 software (QSR International, Cambridge, MA, USA). Two researchers independently developed a coding scheme by identifying, classifying, and labelling the primary patterns in the data from the transcripts. During open coding, the constant comparative approach was used to group the codes into categories (where each category was considered a unit of analysis) and identify themes. Axial coding was then done to look at the interrelationship of categories [37]. The frequency and consistency with which participants indicated categories in the transcripts were used to provide credibility to these categories. We performed a calibration exercise between two reviewers for appropriately classifying themes into categories using Kappa statistics (in NVivo 8), and any disagreements (considered as <90% agreement) were resolved through consensus by a third reviewer. Videos from usability study 2 were viewed by one researcher and coded only for themes related to general and critical incidents. Data from the coded video were used to supplement themes identified by audio transcripts and to corroborate incident log records from direct observation of participants.

### Quantitative data

Quantitative data were analysed using frequency analysis of demographic questions, task accuracy, and frequency and classes of problems encountered; descriptive statistics to calculate proportions and time to completion of tasks (*e.g*., mean time to RAQ completion with standard deviations [SDs]); Likert-scale questions (mean scores with SDs); independent sample *t*-tests for comparing groups for differences in mean time to RAQ completion (with standard errors of the means [SEs]); and a one-way between-groups analysis of variance (ANOVA) to compare the effects of the three input devices on mean time to RAQ completion. Time data were converted from minutes:seconds to total seconds for data entry into the statistical software, and means and SDs were reconverted to minutes:seconds for results tables; means and their 95% confidence intervals (CIs) for comparison groups were converted to minutes. All statistical analyses were carried out using SPSS (Macintosh version 17.0; IBM Corporation, Somers, NY, USA).

Testing-session worksheets and components of the osteoporosis tool were modified and refined according to changes suggested by quantitatively and qualitatively analysed data and retested if findings indicated that significant changes were recommended. The analysis was thus cumulative and iterative, with new versions of the tool components building on proceeding versions. This procedure was continued with the transcripts and data of subsequent usability sessions until themes were saturated.

## Results

### Usability study 1 (BestPROMPT)

Table 1 shows the characteristics of the 11 physicians (9 family physicians and 2 general internists; 46% between 46 and 55 years of age) who participated in the usability study. The mean overall System Usability Scale score was 80.5 (SD 9.5), which indicates a subjective global view of the BestPROMPT as "relatively easy to use" [35].

**Table 1:** Characteristics of physicians who tested the usability of the Best Practice Recommendation Prompt (BestPROMPT) (N = 11)

Characteristic		N (%)
**Gender**		

	Men	5 (45)
	
	Women	6 (55)

**Age range (years)**		

	25 to 35	3 (27)
	
	36 to 45	3 (18)
	
	46 to 55	5 (46)
	
	56 to 65	1 (9)
	
	> 65	0

**Type of physician**		

	Family	9 (82)
	
	General internal medicine	2 (18)

**Years in practice**		

	< 5	2 (18)
	
	5 to 10	2 (18)
	
	11 to 15	2 (18)
	
	16 to 25	4 (36)
	
	> 25	1 (9)

**Type of patient record system**		

	Electronic Health Record (EHR)	0
	
	Paper-based	6 (55)
	
	Partial EHR	5 (45)
	
	Functions performed on the EHR: Diagnostic and lab results (N = 5)	4 (80)

### Usability worksheet results

Data analyses of the semistructured interviews identified three broad categories of themes:

1. Participants' perceptions of the barriers to using the BestPROMPT: 91% of physicians identified lack of time as the biggest barrier to using the sheet in family practice. Some were concerned that patients might not finish the RAQ in time for the visit or that the tool would be problematic in settings with no extra examination rooms. Other identified barriers to using the tool were related to workflow and administrative processes, such as increased clinic staff workload (*e.g*., explaining the tool to patients, alteration of workflow to make the BestPROMPT available at the point of care). About half of the participants were particularly concerned that the tool may disrupt the real reason for the visit and interrupt or delay the care of patients with more serious symptoms (*e.g*., chest pain). Suggestions to overcome the lack of clarity in the Recommendation Box section of the sheet were to highlight the Diagnosis section, to distinguish between the Diagnosis and Treatment Recommendation sections, and to indicate when a bone mineral density test should be repeated.

2. Participants' perceptions of the facilitators to using the BestPROMPT: Features that were perceived as facilitators were the inclusion of a 10-year absolute fracture risk graph to show patients which risk region (low, moderate, high) they fell into, the inclusion of a Justification section for the recommendations, and the provision of the most important information about risk, diagnosis, and treatment on one page. Participants liked the RAQ summary table because it provided an overview of their patients' responses and highlighted their major and minor risk factors. Some thought that this information could be used as a reminder about risk factors that may have been overlooked or forgotten, and to select which patient should have a bone mineral density test or which treatment should be started.

3. Participants' perceptions of using the BestPROMPT at the point of care: Most participants indicated that they would use the tool at the point of care but not necessarily during a standard scheduled visit. Suggestions were to use the sheet during a dedicated visit for osteoporosis or a physical examination, and physicians believed that these options would provide more time to discuss the information with patients. Suggestions to enhance point-of-care use were to ensure that the practice workflow is considered during tool implementation and to enable the wireless printing of the BestPROMPT so it can be available for review by physicians prior to the patient visit.

### Usability study 2 (RAQ)

Nineteen patients (mean age 72 years; 53% women) from the practice of one family physician participated in the usability study (Table 2). Sixty-eight percent of participants indicated previous experience with using a computer, but less than half (47%) reported ever having used the Internet. The first nine participants (47%) tested the RAQ using a stylus pen as the pilot input device. Subsequent patients were alternated between the mouse/keyboard or touch screen. After two alternations of these devices, participants found the touch screen considerably easier to use, so the mouse/keyboard testing was discontinued.

**Table 2:** Characteristics of patients who tested the usability of the Risk Assessment Questionnaire (RAQ) (N = 19)

Characteristic		N (%)	Mean age (years)	Mean time to RAQ completion (minutes:seconds [SD])	Comparison groups	**Mean difference in time to RAQ completion (minutes [95% CI])**^**a**^
**All**			72	6:35 (5:15)		

**Gender**						

	Women	10 (53)	74	5:56 (1:24)	**Women vs. Men**	1.40 (-3.80 to 6.59)
			
	Men	9 (47)	69	7:19 (7:40)		

**Computer use**						

	Yes	13 (68)	72	6:42 (6:19)	**Use vs. no use**	0.36 (-5.27 to 5.98)
			
	No	6 (32)	72	6:21 (1:52)		

**Internet use**						

	Yes	9 (47)	69	7:00 (7:43)	**Use vs. no use**	0.78 (-4.44 to 6.01)
			
	No	10 (53)	75	6:13 (1:26)		

^a^Calculated using independent samples *t *-test.

SD = standard deviation; CI = confidence interval.

### Usability worksheet results

#### Time to RAQ completion

**Figure 1:**
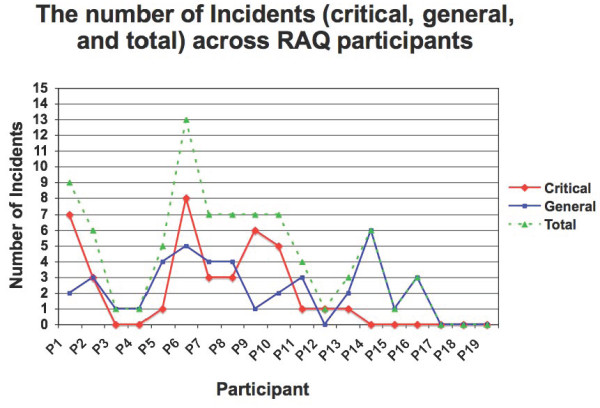
**The number of incidents (critical, general, and total) across participants who tested the Risk Assessment Questionnaire (RAQ)**.

The mean time to RAQ completion was 6:35 (minutes:seconds) (SD 5:15) (Table 2). There was no difference between participants with previous computer use or Internet experience compared with those with no experience for time to RAQ completion (mean difference range 0:22 to 0:47 seconds). Although the mean time to RAQ completion decreased by almost four minutes from initial testing with a stylus pen to the touch screen (Figure 1); a one-way ANOVA analysis showed no significant difference between the three input devices for mean time to RAQ completion (Table 3).

**Table 3:** Characteristics of patients who tested the usability of the Risk Assessment Questionnaire (RAQ) according to three different input devices (N = 19)

Input device		N (%)	Mean age (years)	Mean time to RAQ completion (minutes:seconds [SD])	Comparison groups	**Average difference in time between input devices (minutes) (β [CIs])**^**a**^
	Stylus pen	9 (47)	73	8:27 (7:10)	--	
	
	Mouse/Keyboard	2 (11)	64	6:29 (2:36)	Stylus vs. mouse/keyboard	-1.97 (-10.58 to 6.63)
	
	Touch screen	8 (42)	73	4:31 (1:21)	Stylus vs. touch screen	-3.93 (-9.28 to 1.42)

^a^Calculated using analysis of variance (ANOVA).

*SD = standard deviation; CI = confidence interval.

#### Critical incident analysis

Of 81 incidents observed among 19 participants, 42 were critical and 36 were general incidents (6 general incidents were elevated to critical status). Navigational problems (*i.e*., moving from one RAQ page to the next without assistance) accounted for 60% of the total critical incidents, and 20% of problems were related to input device (*i.e*., mis-tapping, clicking or touching on the tablet PC screen). Most critical incidents (80%) occurred with the first nine participants testing the stylus pen (range zero to eight incidents), but decreased from five incidents (participant 10) to one incident (participants 11 to 13), to no critical incidents observed with the last six participants using the touch screen (Figure 1). Data analysis identified three broad categories of themes from the critical incident log and the semistructured interview of patients:

1. Participants' perceptions of the facilitators to using the RAQ: Fifteen of 19 participants (79%) thought that the questions were clear and simple and easy to read, understand, and use overall. Participants liked the audio feedback and picture aids because these clarified and helped to understand the questions. Of those who tested the touch screen (N = 8), most participants (88%) liked it because it was familiar, even if they had never used a computer: 'It was made easy for me, it was completely natural because it's similar to banking machines, there you've got to touch the screens too, so this reminded me of that'.

2. Participants' perceptions of the barriers to using the RAQ: Several format features impacted use, including the 'Audio' button on the Start page, which many found confusing as it interfered with the successful initiation of the questionnaire. Navigational problems were also identified, including the tendency to unintentionally bypass the second part of two-part questions such as the Periods and Bone Mineral Density pages.

3. Participants' suggestions for improving the RAQ: Suggestions for additional clarity were provided, including creating separate entry fields to distinguish between surname and first name, providing definitions for conditions (*e.g*., rheumatoid arthritis), and providing more direction for participants to move from one page to the next.

### Usability study 3

Eight participants (mean age 76 years; 50% men) from the practice of one family physician participated in this usability study. Of these, seven participants (88%) were recruited from the RAQ usability study sample. The mean time to RAQ completion was 4:31 (minutes:seconds) (SD 1:25), and men completed the RAQ almost two minutes faster than did women (Table 4).

**Table 4:** Characteristics of patients who tested the usability of the Customised Osteoporosis Education (COPE) tool (N = 8)

Characteristic		N (%)	Mean age (years)	Mean time to RAQ completion (minutes:seconds [SD])	Comparison groups	Mean difference in time to RAQ completion (minutes:seconds [SE])	*p *value
**All**			76	4:31 (1:25)			

**Gender**							

	Women	4 (50)	79	5:27 (0:29)	**Women vs. men**	1:52 (0:46)	0.05^a^
				
	Men	4 (50)	72	3:35 (0:26)			

^a^Significant (calculated using independent samples *t*-test).

RAQ = risk assessment questionnaire; SD = standard deviation; SE = standard error.

### Usability session worksheet

Data analysis from the semistructured interview identified two broad categories of themes:

1. Participants' perceptions of what they liked about the COPE sheet overall: Most participants (88%) were able to understand and describe specific sections. When asked what they would do with the COPE sheet, all eight participants indicated that they would discuss the information with their physician.

2. Participants' suggestions for improving the COPE sheet: Several content and formatting suggestions were made, including using simpler language (*e.g*., to modify 'Your responses to the questionnaire' to 'This is your answer') and improving the layout so that the table in the COPE sheet extended all the way to the bottom. The COPE sheet was iteratively changed reflecting these suggestions after the first four participants and after the last participant.

## Discussion

The three components of the osteoporosis tool were evaluated in individual usability studies to determine how well the prototype met end users' needs, functional goals (features, format, content, navigation), and outcome impact goals (*e.g*., the use of the tool at the point of care). Of the three components of the osteoporosis tool that were tested, the RAQ required the most cycles of iteration to meet the needs of patients at risk for osteoporosis, which may be attributed to several factors. First, the format of the RAQ is complex because it is computer-based and interactive, while the other components are paper-based. Since the RAQ is computer-based, it can also support a system for adapting to evolving evidence about osteoporosis disease management. For example, the decision algorithm of the RAQ was originally programmed according to the 2002 osteoporosis guidelines [9] but can be easily updated to reflect changing guidelines. Second, the majority of people that would be targeted to use the RAQ are older (age ≥65 years). This is a population that tends to have less experience with computerised systems and may have motor or cognitive impairments or visual deficiencies that may require more attention to interface design (*e.g*., font and tab size and colour), content (*e.g*., wording and amount of information), and ease of navigability.

**Figure 2:**
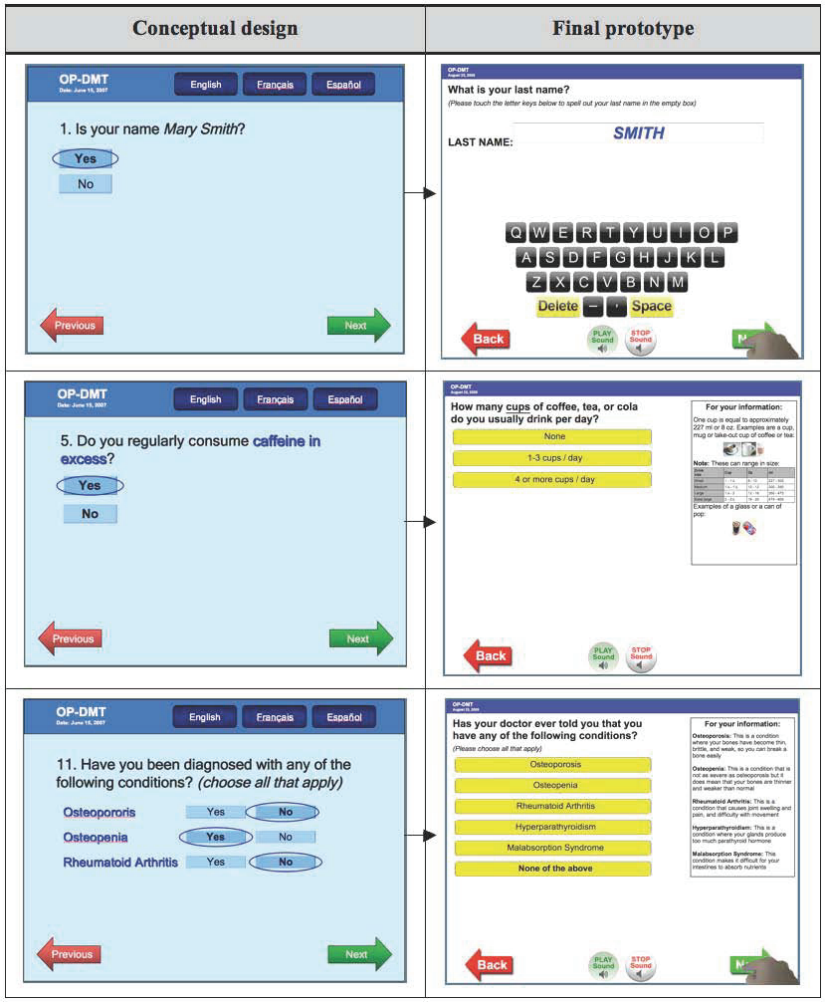
**Screen shots depicting the evolution of selected RAQ questions**.

**Figure 3:**
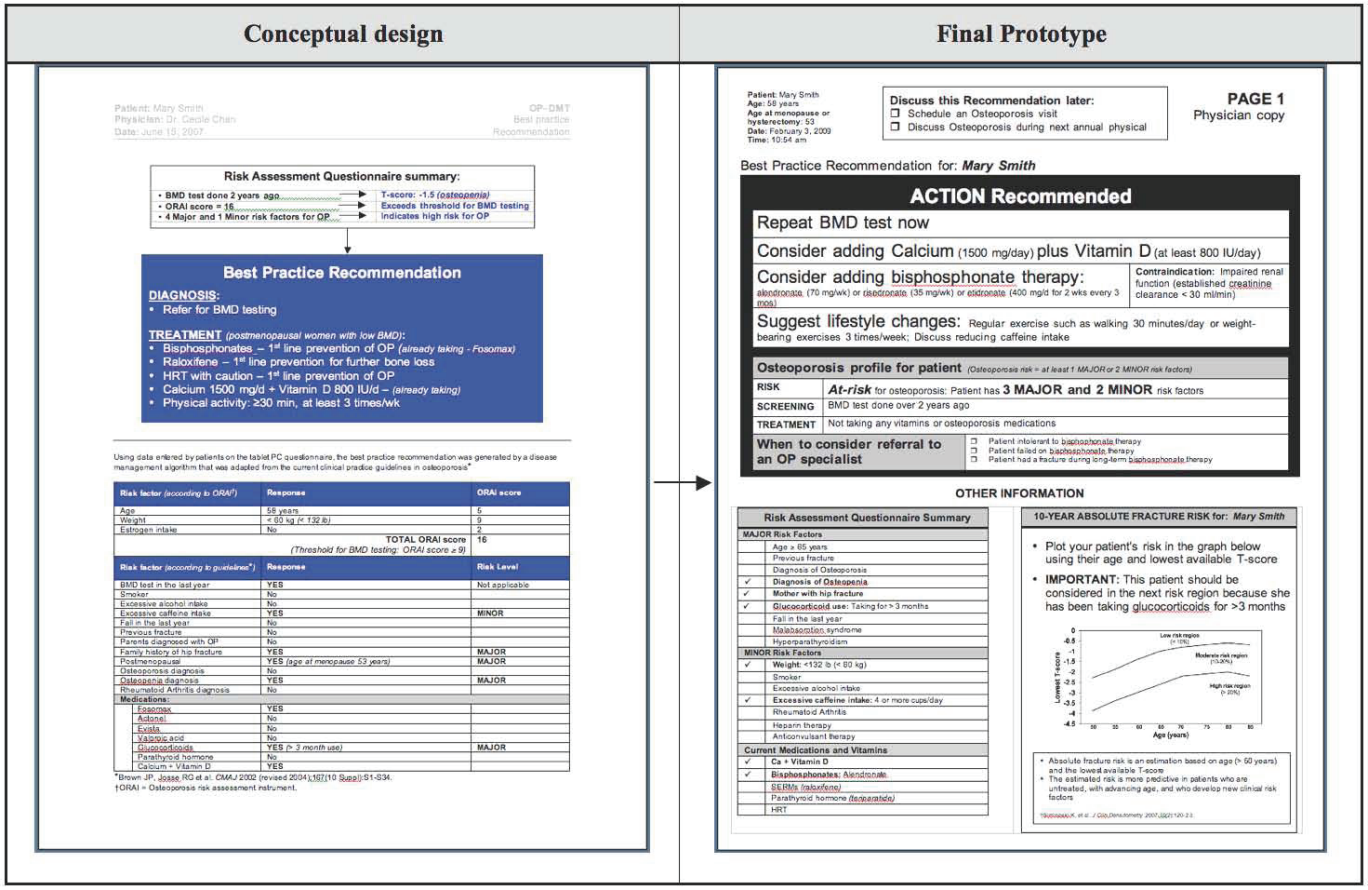
**Screen shots depicting the evolution of the BestPROMPT sheet for physicians**.

**Figure 4:**
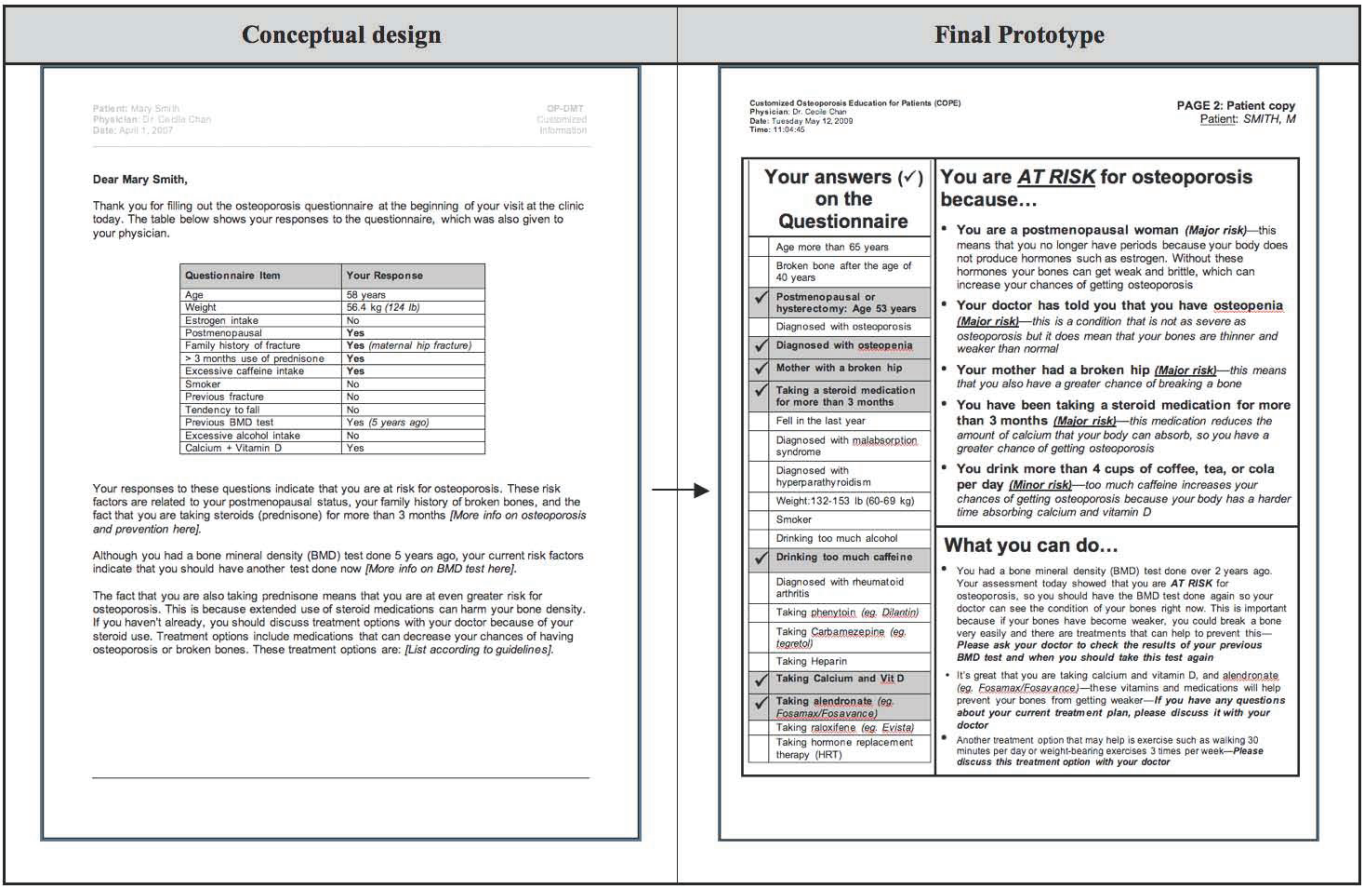
**Screen shots depicting the evolution of the COPE sheet for patients**.

The think-aloud approach enabled the observation of end users as they carried out relevant tasks while interacting with individual tool components. This process was very helpful for identifying specific problems and to iteratively modify the system accordingly. The transformations of the tool from pre- to post-usability prototype are shown in Figure 2 (selected screenshots of the RAQ), Figure 3 (screenshot of the BestPROMPT sheet), and Figure 4 (screenshot of the COPE sheet), and a demonstration of the tool can be accessed at http://knowledgetranslation.ca/osteo_final/.

Several challenges to point-of-care use of CDSSs in family practice emerged from the findings of the usability studies. It is not surprising that physicians indicated lack of time or resources to use the osteoporosis tool as a major barrier to point-of-care use, as this has been identified in other studies investigating CDSSs [20,21]. However, an unexpected barrier also emerged--the osteoporosis tool might unintentionally disrupt the real reason for the visit. Although evidence indicates that providing CDSSs at the point of care may improve clinical practice [21], there are challenges to designing such tools for family practice settings because the physician-patient encounter can be disrupted. Although we achieved the goal of designing a quick and easy tool (*i.e*., the last eight patients completed the RAQ in a mean 4:31 minutes and the last six initiated the questionnaire without assistance), physicians suggested that the provision of osteoporosis information at the point of care could interfere with their usual practice in other ways. First, the practice visit agenda may be disrupted because the experience of working through the RAQ may prompt patients to ask questions about osteoporosis during the visit. Second, the introduction of either the BestPROMPT or COPE sheets can facilitate the transmission of osteoporosis knowledge between provider and patient, but this has to be weighed carefully against the cost of interrupting or halting the discussion of more urgent aspects of the patient's intended visit agenda (*e.g*., chest pain) or health status (*e.g*., diabetes). This finding should be an important consideration when designing point-of-care tools and highlights the need for a flexible and pragmatic approach when planning how such tools should be implemented and used in family practice. Interventions that are adapted to their local settings and are tailored to the specific needs of physicians should be considered for systems to better fit the real practice workflow [24-26,39]. It might also be useful to provide physicians with a choice to either act on or defer the use of point-of-care information, depending on the context of the patient visit. Physicians are more likely to adopt CDSSs if they have some control over the way it is used, without giving up complete autonomy of their clinical decision-making [26,40]. In the case of the osteoporosis tool, this would enable physicians to use information about osteoporosis at their discretion without having to compromise the well-being of their patients or care agenda.

### Limitations

There are a number of limitations to the usability studies. First, although we exceeded our target sample sizes, it is possible that the inclusion of more participants may have uncovered more information or problems or have shown significant differences between comparison groups for time to RAQ completion. Second, we recruited all 19 patients from the patient population of one family physician, and more than half of physicians were recruited from the same inner-city center family practice unit, which may not be representative of other family physicians and their patients or settings. However, given the demographics of the participants, they appear similar to other patients with osteoporosis. Third, we excluded the System Usability Scale questionnaire from patient usability testing, so it was not possible to calculate an overall usability score for either the RAQ or COPE components of the tool. We wanted to optimise the balance between getting feedback about the usability of these tool components without exhausting the mostly elderly participants. Additionally, the recruitment process restricted the opportunity to extend sessions to include the System Usability Scale since most patients were recruited immediately after their family physician appointment, when many patients were too tired, weak, or ill to participate in a study lasting more than 30 minutes. Lastly, control for selection bias was difficult because patients who tested the RAQ and COPE sheet were selected from the same practice setting (*i.e*., the St. Michael's Hospital Family Practice Unit). However, their inclusion was also useful because they were able to see two components of the tool.

## Conclusions

Results from the three usability studies were used to make informed modifications and refinements to the osteoporosis tool prototype. Major challenges to point-of-care use of the tool were physicians' lack of time and that the tool might unintentionally disrupt the real reason for the visit. These challenges indicate that implementation of such tools in family practice requires a flexible and pragmatic approach. The findings also confirm the importance of usability testing of interactive clinical decision support applications and information systems on all end users to reduce problems and errors, particularly if the future goal is to implement such systems in a clinical practice setting. The findings of the usability studies also highlight the need to include usability evaluation as an important step in the development process of knowledge translation interventions.

## Competing interests

The authors declare that they have no competing interests.

## Authors' contributions

All authors participated in the design of the study. MK and CM conducted the usability testing sessions. MK, CM, and SES performed the analysis. MK drafted the manuscript, and all authors read and approved the final manuscript.
